# Brachial plexus lipomatosis with perineurial pseudoonion bulb formation: Result of a mosaic PIK3CA mutation in the para‐axial mesoderm state

**DOI:** 10.1111/bpa.13057

**Published:** 2022-02-27

**Authors:** Blake A. Ebner, Kathryn L. Eschbacher, Megan M. Jack, Milosevic Dragana, Robert J. Spinner, Caterina Giannini

**Affiliations:** ^1^ 6915 Department of Laboratory Medicine and Pathology Mayo Clinic Rochester Minnesota USA; ^2^ 6915 Department of Neurosurgery Mayo Clinic Rochester Minnesota USA

**Keywords:** brachial plexus, lipomatosis of nerve, perineurial cell pseudo‐onion bulb, PIK3CA‐related overgrowth spectrum, segmental overgrowth

## CONFLICT OF INTEREST

All authors have no conflict of interest to report.

## ETHICAL APPROVAL

The study was approved by the Mayo Clinic Institutional Review Board.

## AUTHOR CONTRIBUTIONS

M.J. and R.S. performed the branchial plexus dissection. B.E., K.E., M.J., and C.G. designed the study. B.E. and M.D. performed the PCR experiments and analyzed the results. B.E., K.E., and C.G. analyzed and interpreted the histology. B.E., K.E., M.J., and C.G. wrote the manuscript. All authors participated in reviewing and editing the final version of the manuscript.

Lipomatosis of nerve (LN), previously known as fibrolipomatous hamartoma, is characterized by overgrowth of mature adipose tissue within the epineurium, surrounding and separating nerve fascicles [1] and is often associated with perineurial thickening and endoneurial perineurial cell pseudo‐onion bulb (PPOB) formation [2]. LN is now classified within the PIK3CA‐related overgrowth spectrum (PROS) characterized by segmental (nerve territory) overgrowth of soft tissue and bony structures [1]. PROS disorders are characterized primarily by the type of mesenchymal tissue overgrowth they exhibit (fibroadipose tissue, bone, and vasculature). In addition to LN, they include a variety of syndromes such as CLOVES (congenital lipomatous overgrowth, vascular malformations, epidermal nevi, scoliosis/skeletal, and spinal), CLAPO (capillary malformation of the lower lip, lymphatic malformation of the face and neck, asymmetry of face and limbs, partial or generalized overgrowth), orofacial overgrowth, and others. LN and orofacial overgrowth are the only PROS disorders that demonstrate PPOB formation. Segmental overgrowth, as seen in LN, is thought to be the result of postzygotic mutations occurring early in embryonic development and affecting a subset of downstream somatic cells in two or more genetically distinct cell lineages. As a result, LN has a distinct phenotype with involvement of most major peripheral nerves and a high degree of variability in disease severity. While LN has predilection for certain nerve territories (median), what mechanism drives the nerves involved or severity is not well understood. Likely responsible for the phenotype restricted to a single‐limb territory are heterozygous somatic activating mutations of the phosphatidylinositol 3‐kinase (*PIK3CA*) gene [3] occurring in a post‐zygotic (somatic) subset of mesodermal cells involved in development of a portion of limb tissues, including adipose tissue. The nature of the accompanying PPOB, if hyperplastic/reactive or part of the lesion, remains at present indeterminate [4].

**FIGURE 1:**
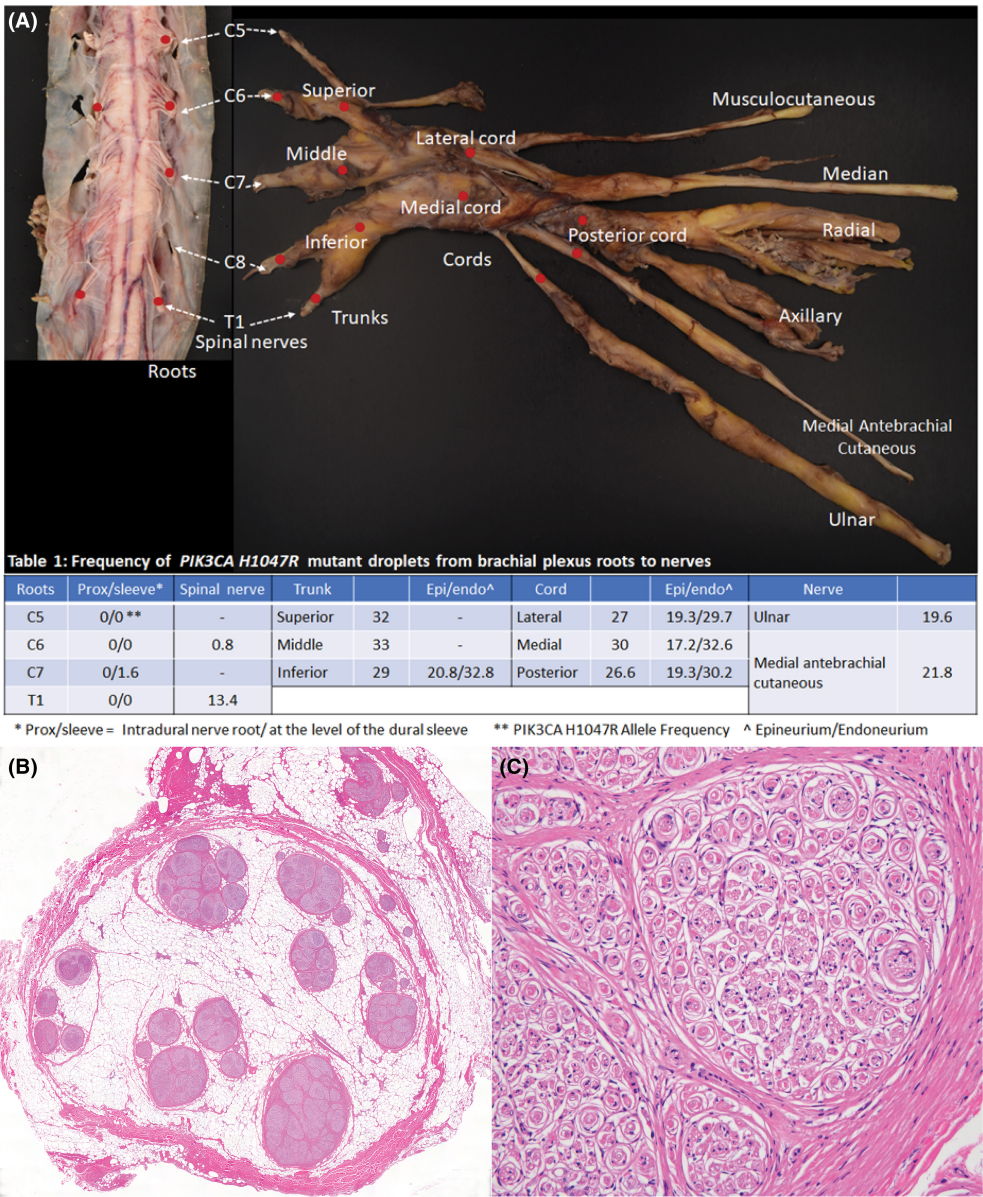
(A) Gross image of spinal cord and left brachial plexus demonstrating diffuse enlargement. Red dots indicate the site tested for PIK3CA mutation by digital droplet PCR (ddPCR). Table 1 lists *PIK3CA H1047R* allele relative frequency per brachial plexus segment (%). Intradural and dural sleeve components were separated at the roots (C5, C6, C7 and T1). Epineurium and endoneurium compartments were isolated from inferior trunk and cords. (B) Representative H&E images of the medial cord demonstrating extensive adipose infiltration of the epineurium separating nerve fascicles. (C) High magnification of square inset with PPOB formation

We present the autopsy findings from a woman with extensive LN of the brachial plexus with associated nerve‐territory overgrowth and macrodactyly. The patient's clinical findings have previously been described in detail [5]. Briefly, the patient is a 55‐year‐old female with a history of progressive congenital left arm overgrowth including macrodactyly of the 3^rd^ and 4^th^ digits. Limited family history was known due to adoption. The patient had multiple surgical debulking's of the overgrowth, the current mainstay treatment. The enlargement severely restricted the patient's arm function and hand dexterity. Later in life, the patient developed paresthesias of the thumb and index fingers and the dorsum of the hand and forearm. MRI of the left shoulder had shown characteristic features of LN, including enlarged infraclavicular brachial plexus with prominent interdigitating adipose surrounding and splaying nerve fascicles. The patient had been followed at our institution and unfortunately died unexpectedly. The autopsy examination showed the brachial plexus was diffusely affected, beginning very proximally from the spinal nerves and extending distally to the major limb nerves (Figure 1A). Histologically, extensive adipose infiltration of the epineurium separating nerve fascicles was found. Variable but extensive PPOB formation was present involving the entire brachial plexus including proximal spinal nerves (just distal to the dural sleeve), trunks and cords, and major terminal branches in the axilla (Figure 1B,C).

*PIK3CA*, *H1047R* (c.3140A > G, p. His1047Arg), a recognized hotspot mutation both in PROS and LN [1] was identified at the level of the brachial plexus trunks, cords, proximal ulnar nerve, and medial antebrachial cutaneous nerve using digital droplet PCR (ddPCR) (Figure 1). At four distinct areas within the brachial plexus, we were able to isolate DNA separately from the epineurium containing abundant adipose tissue, vessels and fibroblasts, and nerve fascicles with extensive PPOB, in which perineurial cells and Schwann cells ensheathing axons predominate (Figure 1A). The *PIK3CA H1047R* mutation was present in both compartments, with the respective frequency of 39.1% (number of *H1047R* mutant droplets/number of wild‐type plus *H1047R* mutant *PIK3CA* droplets) in the endoneurium and 19.1% in the adipose tissue rich epineurium (Figure 1).

As the *PIK3CA H1047R* mutation was present in both compartments, we hypothesized the mutation‐driving LN in this individual may have occurred in early embryonic development and affected a common precursor of both adipose tissue and perineurial cells, the two elements primarily involved in the overgrowth. While the origin of perineurial cells remains under investigation, one hypothesis is that they may derive from specialized mesodermal fibroblasts, at the level of the para‐axial mesoderm, together with adipocytes and fibroblasts during the early pre‐somitic or early somitic mesoderm state (20–30 days) [6]. Our findings would be in keeping with this hypothesis.

To test this hypothesis, we assessed separately the nerve roots just inside and at the level of the dural sleeve for the presence of the *PIK3CA H1047R* mutation (Figure S1A–D), where sensory and motor nerve fibers blend to become a spinal nerve and where the transition occurs from the dura to the mesoderm‐derived epineurium and perineurium. Proximal to the sleeve, only neuroectodermal (axons) and neural crest (Schwann cells, pia/dura)‐derived elements are present. Histologic examination of the roots inside the dura did not show LN involvement. At the level of the dural sleeve, C5‐C6 and C8 spinal nerves did not show any obvious abnormality, while C7 demonstrated minimal hypercellularity. Just distal to the sleeve, there were focal but obvious PPOB in the T1 spinal nerve (Figure S2). The *PIK3CA H1047R* mutation was absent intradurally. Immediately distal to the sleeve, a single spinal nerve (C7) was positive for the *PIK3CA H1047R* mutation at a very low allele frequency of 1.6%. When we assessed the spinal nerves distally to these initially tested segments, we also identified the *PIK3CA H1047R* mutation (C6 0.8%, C8 1.0%, T1 13.4%) (Figure S2). Two contralateral nerve roots were also tested (C6 and T1) and were negative.

This study demonstrates the presence of the *PIK3CA* mutation in both the brachial plexus adipose‐rich epineurium and in the endoneurium where PPOB predominates. These findings, in conjunction with the absence of intradural *PIK3CA H1047R* mutation, suggest that the mutation in both the epineurium and endoneurium involves predominantly mesodermal‐derived distinct cell types, including adipocytes and perineurial cells. These findings support the hypothesis that perineurial cells are derived from the mesoderm. Historically, perineural cells were thought to be derived from specialized fibroblasts based upon their ultrastructural characteristics in fetal development, in addition to in vitro studies utilizing fibroblasts and Schwann cells infected with retrovirus [7]. Our findings are in contrast with the alternative hypothesis of a neuroectodermal origin of perineurium recently suggested by a study utilizing transgenic zebrafish, which found some ventral spinal cord cells migrating in a chain‐like manner down outgrowing motor axons to resemble ultrastructurally perineural cells [6]. Resolving this discrepancy will require further investigation. Our results are in keeping with the presence of *PIK3CA* mutation in perineurial cells and supports the view that the formation of PPOB, which so closely resemble PPOB seen in intraneural perineurioma [8], is part of the LN process rather than simply a hyperplastic reaction to the extensive adipose tissue deposition. Histologically similar endoneurial changes have been recently reported as “perineuriomatous pseudo‐onion bulb proliferations” in orofacial overgrowth, which has also been shown to harbor *PIK3CA* mutations [9].

We postulate that in our case the *PIK3CA* mutation originated in early mesodermal progenitors that eventually migrated from the limb bud to form their respective tissues. Our results suggest the origin of PIK3CA mutation in this case is likely at the dural junction/limb bud and results in both perineurial cell and adipocytes maintaining the PIK3CA mutation.

This case of LN with localized overgrowth of the brachial plexus territory provides insight into the timing, distribution, and cells housing the postzygotic PIK3CA mutation established early in embryonic development. Indeed, other syndromes part of the PIK3CA‐related overgrowth spectrum, such as fibroadipose hyperplasia, also demonstrate diffuse segmental lipomatosis overgrowth but without involvement of the perineurial compartment, suggesting a non‐perineurial precursor cell type involved by the mutation. Thus, this supports the hypothesis that the differentiating factor between the PROS spectrum disorders is the fate of the cell type originally affected.

A long‐standing question has been why LN does not involve the central nervous system [10]. The absence of detectable *PIK3CA* mutation intradurally is in keeping with the view that in LN *PIK3CA* mutation does not occur in cells of neuroectodermal or neural crest origin and, therefore, this compartment cannot be involved.

## 1 METHODS

The study was approved by the Mayo Clinic Institutional Review Board. Slides used were fixed in neutral buffered formalin and embedded in paraffin for histologic processing. The paraffin‐embedded tissue blocks were used for both conventional hematoxylin–eosin stain and for DNA extraction.

### 1.1 DNA extraction

DNA from unstained slides was isolated using QIAmp DSP DNA Formalin Fixed Paraffin Embedded Tissue kit (Qiagen, Valencia, CA) as per manufacturer's instructions.

### 1.2 Droplet digital polymerase chain reaction assay (ddPCR)

DNA extracted from different tissue sections was initially tested for the presence of the most common PIK3CA mutations (p.H1047R, p.H1047L, p.E545K, p.E542K). Primers for ddPCR were previously described [1]. Commercial Taqman assays for detection of the PIK3CA H1047R mutation were purchased from BioRad. Reaction mixtures were prepared in 22ul volume in 96‐well plates. The plates are sealed with aluminum foil and centrifuged at 1000 *g* for 1 min and placed on an automated droplet generator (AutoDG, Bio‐Rad). PCR amplification was than performed on a Veriti Thermal Cycler (Applied Biosystems): 95°C for 10 min, then 40 cycles for denaturation at 94°C for 30 s and annealing/extension at 55°C for 1 min, and a final enzyme deactivation at 98°C for 10 min. The completed reactions were stored at 4°C until analysis on a QX200 droplet reader (Bio‐Rad). Total number of mutant and wild‐type copies for each sample were quantified using QuantaSoft software (BioRad Laboratories). The percentage of the mutant allele frequency was calculated by dividing the number of mutant droplets by the number of PIKCA‐wild type plus mutant droplets.

## Supporting information


**FIGURE S1** (A and B) Representative H&E images of the of the C7 intradural and dural sleeve components. Dotted outline depicts the area isolated by microdissection for analysis by ddPCR. (C and D) Insets with high magnification (20x) images of respective nerve fibers
**FIGURE S2** Representative H&E images (20x) of the spinal roots, C6, C7, and T1. Focal PSOB was observed at the level of the root in T1

## Data Availability

Data sharing not applicable to this article as no datasets were generated or analyzed during the current study.
