# Supplementary material for: Brachial plexus lipomatosis with perineurial pseudoonion bulb formation: Result of a mosaic PIK3CA mutation in the para‐axial mesoderm state
Source: Brain Pathol. 2022 Feb 27;32(4):e13057. doi: 10.1111/bpa.13057 (PMC9245934; doi:10.1111/bpa.13057)
Supplement: Supplementary file 1 — FIGURE S1 (A and B) Representative H&E images of the of the C7 intradural and dural sleeve components. Dotted outline depicts the area isolated by microdissection for analysis by ddPCR. (C and D) Insets with high magnification (20x) images of respective nerve fibers FIGURE S2 Representative H&E images (20x) of the spinal roots, C6, C7, and T1. Focal PSOB was observed at the level of the root in T1 [file BPA-32-e13057-s001.pdf]

Brachial plexus lipomatosis with perineurial pseudoonion bulb formation: result of a mosaic PIK3CA mutation in the para-axial mesoderm state?

Blake A. Ebner M.D., Ph.D[1], Kathryn Eschbacher M.D. [1], Megan M. Jack M.D., Ph.D[2], Milosevic Dragana M.S. [1], Robert J. Spinner, M.D. [2], Caterina Giannini M.D., Ph.D[1*].

[1] Department of Laboratory Medicine and Pathology and [2] Department of Neurosurgery, Mayo Clinic, Rochester (MN), USA

*Ebner.blake@mayo.edu

Supplemental Materials

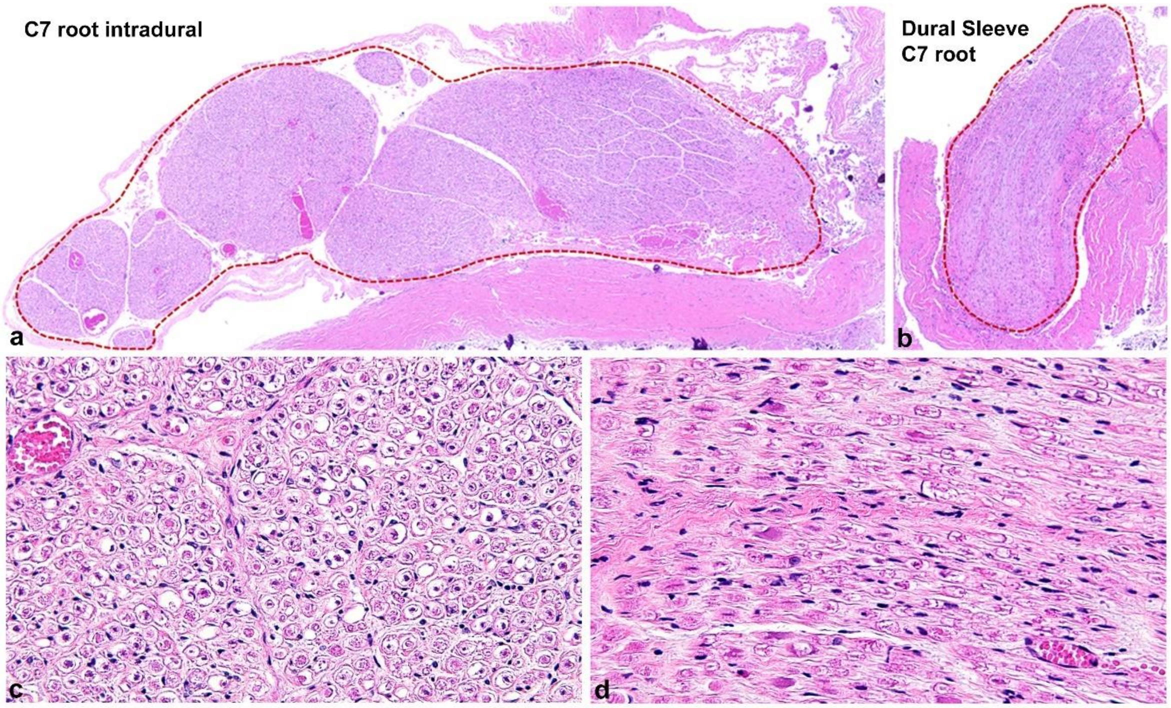

Supplemental Figure 1: (**a, b**) Representative H&E images of the of the C7 intradural and dural sleeve components. Dotted outline depicts the area isolated by microdissection for analysis by ddPCR. **(c, d)** Insets with high magnification (20x) images of respective nerve fibers.

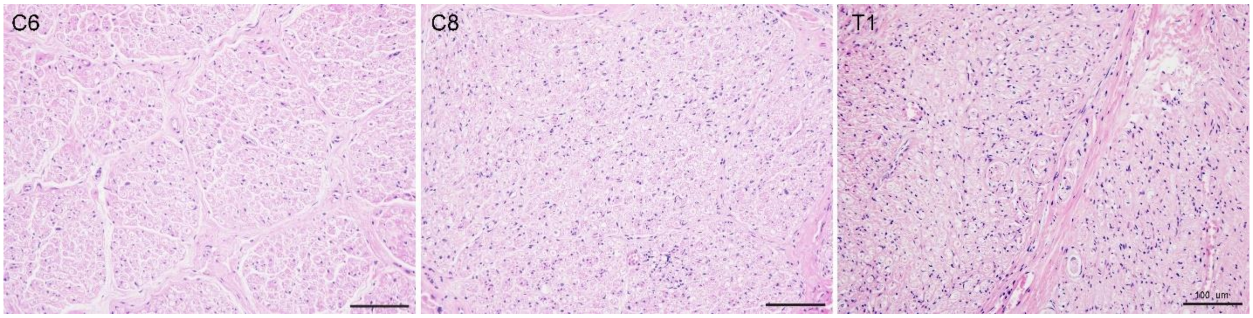

Supplemental Figure 2: Representative H&E images (20x) of the spinal roots, C6, C7, and T1. Focal PSOB was observed at the level of the root in T1.
